# Symbiotic Algae of *Hydra viridissima* Play a Key Role in Maintaining Homeostatic Bacterial Colonization

**DOI:** 10.3389/fmicb.2022.869666

**Published:** 2022-06-06

**Authors:** Jay Bathia, Katja Schröder, Sebastian Fraune, Tim Lachnit, Philip Rosenstiel, Thomas C. G. Bosch

**Affiliations:** ^1^Institute for Zoology and Organismic Interactions, Heinrich Heine University Düsseldorf, Düsseldorf, Germany; ^2^Zoological Institute, Kiel University, Kiel, Germany; ^3^Department of Anatomy, Kiel University, Kiel, Germany; ^4^Institute of Clinical Molecular Biology (IKMB), Kiel University, Kiel, Germany

**Keywords:** symbiosis, microbiome, tripartite interactions, horizontal transmission, co-cultivation

## Abstract

The freshwater polyp *Hydra viridissima* (*H. viridissima*) harbors endosymbiotic *Chlorella* algae in addition to a species-specific microbiome. The molecular basis of the symbiosis between *Hydra* and *Chlorella* has been characterized to be metabolic in nature. Here, we studied the interaction between the extracellularly located microbiota and the algal photobiont, which resides in *Hydra*’s endodermal epithelium, with main focus on *Legionella* bacterium. We aimed at evaluating the influence of the symbiotic algae on microbial colonization and in shaping the host microbiome. We report that the microbiome composition of symbiotic and aposymbiotic (algae free) *H. viridissima* is significantly different and dominated by *Legionella spp. Hvir* in aposymbiotic animals. Co-cultivation of these animals resulted in horizontal transmission of *Legionella spp. Hvir* bacteria from aposymbiotic to symbiotic animals. Acquisition of this bacterium increased the release of algae into ambient water. From there, algae could subsequently be taken up again by the aposymbiotic animals. The presence of algal symbionts had negative impact on *Legionella spp. Hvir* and resulted in a decrease of the relative abundance of this bacterium. Prolonged co-cultivation ultimately resulted in the disappearance of the *Legionella spp. Hvir* bacterium from the *Hydra* tissue. Our observations suggest an important role of the photobiont in controlling an invasive species in a metacommunity and, thereby, shaping the microbiome.

## Introduction

**FIGURE 1:**
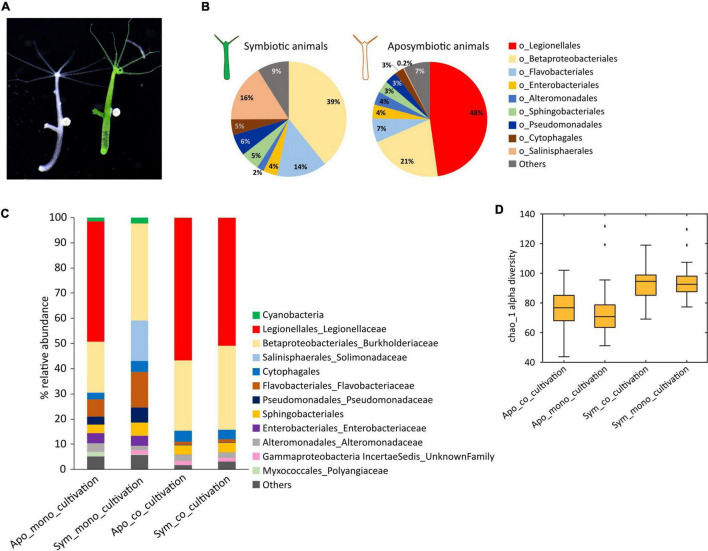
Co-cultivation affects the microbiome composition of symbiotic and aposymbiotic *Hydra viridissima* (*H. viridissima*) polyps. **(A)** Symbiotic (green) and aposymbiotic (white) *Hydra viridissima* polyps. **(B)** Pie charts showing the mean relative abundances (*N* = 58) of bacteria at order level of symbiotic and aposymbiotic *H. viridissima*. **(C)** Taxonomic bar charts representing mean relative abundances of bacteria in co-cultivated and monocultivated symbiotic and aposymbiotic *H. viridissima*. Co-cultivation of symbiotic with aposymbiotic animals drastically affects the microbiome of symbiotic animals, in particular due to the acquisition of a single operational taxonomic unit (OTU), *Legionella spp. Hvir*. **(D)** Box plots showing the alpha-diversity (Chao1) of symbiotic and aposymbiotic polyps under mono- and co-cultivation.

For over half a century, *Hydra viridissima* (*H. viridissima*) has been a subject of study for symbiosis between the host polyp and the endosymbiotic *Chlorella* algae (Muscatine and Lenhoff, 1963, 1965; Muscatine et al., 1975; McAuley, 1981a,b; Thorington and Margulis, 1981; McAuley and Darrah, 1990; Figure 1A). We have shown previously that the mutual exchange of metabolites forms the basis of this symbiotic relationship (Hamada et al., 2018). The algae provide fixed carbon in the form of maltose to the host that provides a significant competitive advantage compared to the aposymbiotic animals during periods of starvation, promoting oogenesis, and allow a faster population growth rate (Habetha et al., 2003; Hamada et al., 2018). Symbiotic algae have also been shown to protect the host under temperature stress conditions (Ye et al., 2019a,b). A hallmark of this strong interdependence is the loss of algal autonomy, as it must depend on the host for survival. Genomic analysis of *Chlorella A99* revealed degeneracy in the nitrate assimilation pathway that renders it to depend on host-derived glutamine as the source of nitrogen (Hamada et al., 2018). As a consequence, all the attempts to cultivate these symbiotic algae *in vitro* have failed so far (Hamada et al., 2018).

Apart from the symbiotic algae, the green *H. viridissima* also harbors a distinct bacterial community (Franzenburg et al., 2013b). The bacterial composition for any given *Hydra* species is specific and mirrors the phylogenetic relationship of their hosts (Fraune and Bosch, 2007; Franzenburg et al., 2013b), a pattern termed as phylosymbiosis (Brucker and Bordenstein, 2013). Species-specific antimicrobial peptides and stem cell transcription factor FoxO are involved in shaping *Hydra*’s microbiota, which is remarkably stable over time (Fraune and Bosch, 2007; Franzenburg et al., 2013a; Augustin et al., 2017; Mortzfeld et al., 2018; Bosch and Zasloff, 2021). *Hydra*-associated bacteria protect the host against fungal pathogen infection (Fraune et al., 2015) and influence the host behavior (Murillo-Rincon et al., 2017; Klimovich et al., 2020). In *Hydra*, the bacterial symbionts are known to be vertically transmitted through egg after sexual reproduction following a temporal pattern of colonization (Franzenburg et al., 2013a) or *via* budding from parent polyp. However, the role of horizontal transmission between individuals or through environment is understudied. Moreover, it is unclear yet how in *H. viridissima*, the host, endosymbiotic algae, and the extracellular bacteria impact each other and interact to maintain a tripartite relationship.

Here, we wondered if the alga had any influence on the microbial composition of the polyp. We used *H. viridissima* strain A99 associated with its native *Chlorella A99* symbiont and species-specific bacteria and compared its microbiome with that of the aposymbiotic animals. We tested the role of horizontal transmission of bacteria and other factors of the co-cultivated hosts in determining the microbiome composition. We also tested the role of algal symbionts in shaping the composition of the host-associated microbiome under different cultivation conditions. Our primary focus member of microbiome is *Legionella spp. Hvir*, as we observed clear dynamics in the proportion of this bacterium from being present in trace amounts to be the main colonizer in the symbiotic animals. We could observe the impact of horizontally transmitted bacteria, specifically *Legionella spp. Hvir*, on the host fitness and the effect of eventual spread of *Chlorella* photobiont on the control of *Legionella* bacterium in the metacommunity.

## Results

### Microbiome Composition of Symbiotic and Aposymbiotic *Hydra viridissima* Is Distinctly Different

Symbiotic and aposymbiotic *H. viridissima* polyps maintain a profoundly different microbiome [Adonis, *R*^2^ = 0.35679, *p*-value (Bonferroni corrected) = 0.005] (Figure 1B). While the microbiome of symbiotic animals is dominated by Betaproteobacteriales, aposymbiotic polyps are mostly colonized by bacteria belonging to the Legionellales group (Figure 1B). The prevalent operational taxonomic unit (OTU) in aposymbiotic polyps belongs to the genus *Legionella* (henceforth *Legionella spp. Hvir*). In the symbiotic animals, this microbe is present only in very small numbers (Figure 1C and Supplementary Figure 1). Since aposymbiotic animals were generated from symbiotic *H. viridissima* polyps (see Section “Materials and Methods”), they are genetically identical and we suspected that the absence of *Chlorella* algae in aposymbiotic polyps was responsible for the observed difference in the microbiome composition. This assumption is supported by the observation that the Chao1 index for alpha-diversity was higher for the symbiotic animals as compared to the aposymbiotic polyps, indicating a loss of low abundance taxa in the aposymbiotic animals (Figure 1D; Apo_mono_cultivation vs. Sym_mono_cultivation, *t*-test, *p*-value < 0.01).

### Co-cultivation Affects the Microbiome Composition of Symbiotic and Aposymbiotic *Hydra viridissima*

While *Legionella spp. Hvir* is present in very low numbers (0.03%) in symbiotic *H. viridissima* polyps, it accounts for up to 47.8% of the microbiome in the aposymbiotic animals. When cultured separately, both the *H. viridissima* lines maintain these distinct microbiome signatures indefinitely. However, co-cultivating the two lines for 4 weeks (Figure 1C) resulted in a clear shift of the microbiota in the symbiotic animals and a significant increase of the average relative abundance of *Legionella spp. Hvir*. In symbiotic *H. viridissima* polyps co-cultivated with aposymbiotic polyps, *Legionella spp. Hvir* accounted for up to 50.9% of associated bacteria. This increase in the *Legionella spp. Hvir* abundance was consistent across all the samples of the co-cultivated symbiotic animals (Supplementary Table 1). We presume that this is due to co-housing and the horizontal transfer of *Legionella spp. Hvir* bacteria from aposymbiotic to symbiotic polyps. Despite this shift in microbial composition, it was noteworthy that the Chao1 alpha-diversity index remained higher for the symbiotic polyps as compared to the aposymbiotic animals (Figure 1D; Apo_co_cultivation vs. Sym_co _cultivation, *t*-test, *p*-value < 0.01; Apo_co_cultivation vs. Apo_mono_cultivation, *t*-test, *p*-value > 0.05). This portrays the role of *Chlorella* symbiont in maintaining the diversity of bacteria on host.

### *Legionella spp. Hvir* Can Be Transmitted Through Water

**FIGURE 2:**
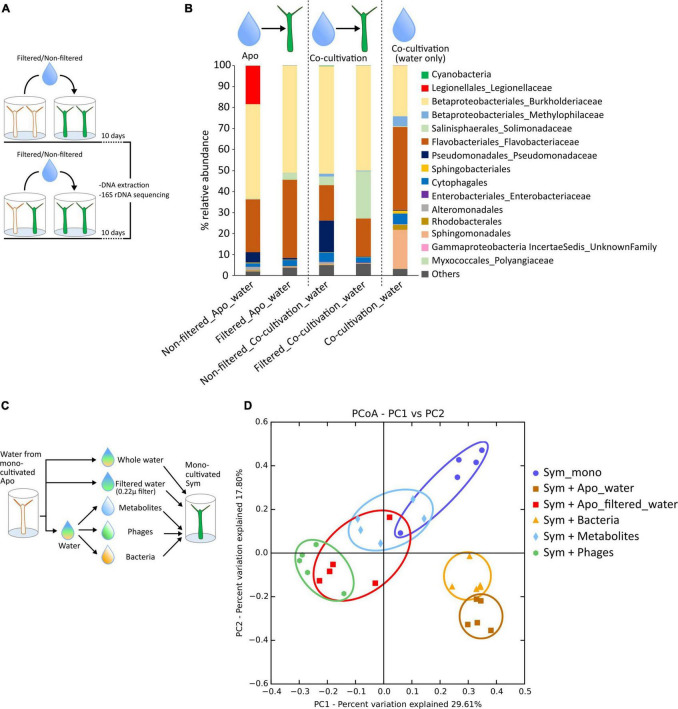
*Legionella* is transferable through water. **(A)** Experimental setup to study the horizontal transfer of *Legionella spp. Hvir*. The mono-cultivated symbiotic animals were incubated in water from the aposymbiotic and co-cultivated animals for 10 days. The water filtered with 0.2 μ filter was used as control. **(B)** Taxa summary plots showing microbiome composition of the symbiotic animals treated with filtered/non-filtered water from aposymbiotic animals and co-cultivated animals along with microbiome composition of water of co-cultivated animals. **(C)** Experimental setup to study the effect of different fractions of water of aposymbiotic animals on the symbiotic animals. **(D)** Principal coordinate analysis (PCoA) plot constructed from Bray–Curtis dissimilarity matrix showing the microbiome composition of the symbiotic animals. The ellipses are manually drawn for visual representation.

To find out how, during the period of co-cultivation, the bacterium spread from aposymbiotic *H. viridissima* to the symbiotic polyps, we next explored whether we could transfer the bacteria simply by exchanging the culture medium and exposing monocultivated symbiotic polyps to water taken from the aposymbiotic culture. As shown in Figures 2A,B, culturing symbiotic animals in the non-filtered water from aposymbiotic animals lead to the presence of a considerable proportion (∼20%) of *Legionella spp. Hvir* in symbiotic *H. viridissima* polyps. Since the co-cultivated culture (4 weeks) also had both the strain of animals colonized with *Legionella spp. Hvir*, we decided to check if the transfer of bacterium can also occurs through the co-cultivation medium. Interestingly and also shown in Figure 2B, the transfer of bacterium only happened when culture medium was taken from an aposymbiotic culture, but not from co-cultivated culture. Consistently, the water from a co-cultivated culture also did not contain any *Legionella* bacteria (Figure 2B). We conclude that *Legionella* from aposymbiotic polyps is transmitted *via* water only in a purely aposymbiotic culture or immediately after the start of the co-cultivation experiment. Moreover, this migration of *Legionella spp. Hvir* is directional toward the symbiotic animals (Supplementary Figure 2). In a long-term co-cultivation experiment, *Legionella* bacteria do not seem to be able to leave the aposymbiotic polyps or the symbiotic polyps anymore.

### Non-bacterial Fractions of Water Affect the Microbiome Composition

The filter-sterilized (with 0.2 μ filter) culture water of the aposymbiotic animals did not contain any bacteria; however, it still resulted in an alteration in the microbiome composition of the symbiotic animals (Figure 2B—Filtered_apo_water) as compared to the native composition of monocultivated symbiotic animals (Figure 1C—Sym_mono_cultivation). To identify the contributing factor responsible for this alteration, we divided the water into the fractions containing bacteria, viruses, and metabolites before treating the symbiotic animals (Figure 2C and Section “Materials and Methods”). It was noteworthy to observe that the virus fraction had a huge impact on the shift in the microbiome composition of the symbiotic animals (Figure 2D) probably through action of phages. A similar trend was also observed with the metabolite fraction. Expectedly, the bacterial fraction had a similar effect as the complete culture water from the aposymbiotic animals, with the main contributing factor being an increase in the *Legionella spp. Hvir* relative abundance (Supplementary Figure 3; For detailed statistics, please refer Supplementary Table 3).

### Transfer of Microbes Influences the Host Fitness

**FIGURE 3:**
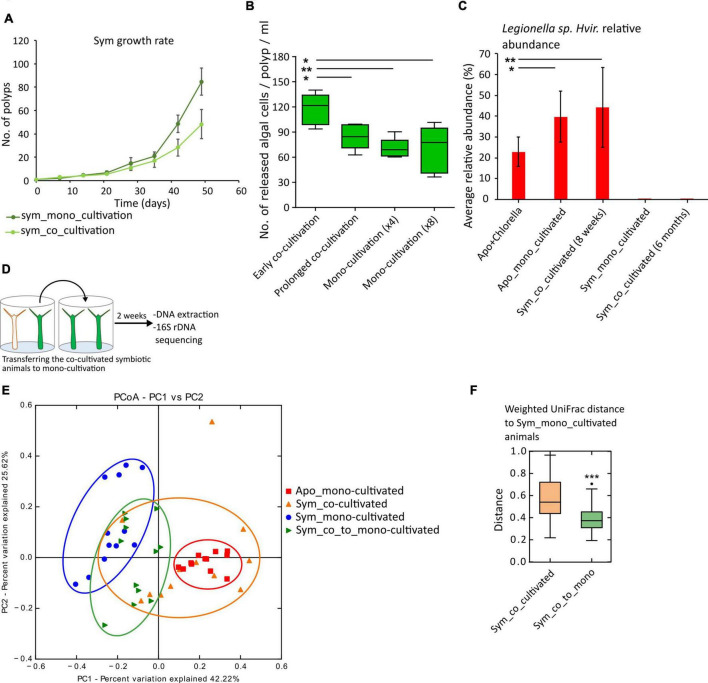
Effect of co-cultivation on the host fitness and microbiome dynamics under co-/mono-cultivation. **(A)** Population growth rate, as a fitness measure of the mono- and co-cultivated symbiotic animals, was lower for co-cultivated animals. **(B)** Immediate early acquisition of *Legionella spp. Hvir* results in an increased expulsion of algae as compared to prolonged co-cultivation and monocultivation of symbiotic animals (*n* = 5, *t*-test, *p-value < 0.05, **p < 0.01). **(C)** Bar chart showing the effect of algal acquisition on *Legionella spp. Hvir* in aposymbiotic animals and prolonged co-cultivation for 6 months (*n* = 6, *t*-test, *p < 0.05, **p < 0.01). **(D–F)** Effect of transferring the co-cultivated animals to monocultivation. **(D)** Experimental setup. **(E)** Principal component analysis (PCA) plot showing the microbiome composition of the symbiotic animals under mono- and co-cultivation and 2 weeks post-transfer from co- to monocultivation and aposymbiotic animals. **(F)** Box plots comparing the weighted UniFrac distance between symbiotic animals under co-cultivation and post-transfer to monocultivation to monocultivated symbiotic animals.

An altered microbiome composition often has an impact on the host (Tiffany and Bäumler, 2019). When assayed for fitness alteration, we observed that the acquisition of *Legionella spp. Hvir* reduced the population growth rates in the co-cultivated symbiotic animals (Figure 3A). Moreover, a similar effect is also observed in aposymbiotic animals, which upon co-cultivation displayed a reduced population growth rate. However, the reason for the observed growth rate in aposymbiotic animals can rather be correlated to the colonization by the algal symbionts than the altered bacterial composition (Supplementary Figure 4).

The symbiotic animals release a small number of algae in the surrounding water, but they tend to release an increased number of algal symbionts in response to stress (McAuley, 1981b), also observed during coral bleaching events. Since *Legionella spp. Hvir* is fitness costly for the host (Figure 3A), we investigated its effect on the algal release in the symbiotic animals by incubating an equal number of symbiotic polyps with aposymbiotic polyps in an equal amount of sterile culture water for 24 h (see Section “Materials and Methods” and Supplementary Figure 5). During the early phase of co-cultivation (4 days post co-cultivation), we observed an increased level of algal release per polyp as compared to monocultivated and long-term co-cultivated (8 weeks) animals (Figure 3B). We predict that the early colonization of host by *Legionella spp. Hvir* induces stress in the polyp triggering an increased release of algal symbionts in the ambient water.

### Acquisition of *Chlorella* and Prolonged Co-cultivation Negatively Affect *Legionella spp. Hvir* Relative Abundance

The released algae by the symbiotic animals can also act as a means of horizontal transfer of the symbionts (Miyokawa et al., 2018). These cells can be taken up by the aposymbiotic animals and can be converted to symbiotic animals. To study the effect of these acquired symbiotic algae on the *Legionella spp. Hvir*, we fed the aposymbiotic animals with the freshly extracted symbiotic algae and observed the microbial composition of the newly formed symbiotic animals. After 8 weeks, the relative abundance of *Legionella spp. Hvir* reduced in the aposymbiotic animals fed with algae as compared to the controls (Figure 3C). Moreover, a long-term co-cultivation for over 6 months resulted in acquisition of algae by all the aposymbiotic animals rendering them to symbiotic state. It further leads to a reduction in the relative abundance of *Legionella spp. Hvir* in the population to undetectable levels in 16S rRNA gene sequencing (Figure 3C).

### Shifting the Co-cultivated Animals to Monocultivation Restores the Microbiome

If the shift observed in the co-cultivated symbiotic animals is only subjective to co-cultivation with the aposymbiotic animals, then removal from co-cultivation should result in the restoration of the microbial composition. Therefore, the 8-week co-cultivated symbiotic animals were transferred to monocultivation (Figure 3D) for 2 weeks and the microbiome composition was compared to the mono- and co-cultivated animals. As shown in Figures 3E,F, the weighted UniFrac distance between the monocultivated animals and the animals removed from the co-cultivation significantly reduced (Figure 3F, ^***^p-value < 0.001) as compared to the co-cultivated symbiotic animals. The detailed statistical results of pairwise comparison of all the treatments using Adonis test with weighted UniFrac distances as input are given in Supplementary Table 4. This indicated the restoration of the native/homeostatic symbiotic microbiome.

## Discussion

Symbiosis study traditionally focused on bipartite host–microbe interactions such as the plant root–*Rhizobium* symbiosis or algae–fungi symbiosis in lichen, while tripartite and multipartite associations received lesser attention. However, it is becoming evident that long-term symbiotic persistence is prevalent not only as two-party, but also as more complex multipartite systems. This study demonstrates that in the green *H. viridissima* species, the photosynthetic *Chlorella* symbiont plays an important role in stabilizing the host bacterial community. Culturing aposymbiotic and symbiotic polyps in a shared environment resulted in horizontal transmission of bacteria from aposymbiotic animals to symbiotic polyps and a dramatic alteration of the microbiome in symbiotic animals. The main driver of alteration in the microbial community of symbiotic animals was identified to be a single bacterial OTU, *Legionella spp. Hvir*. *Hydra* is a freshwater organism and *Legionella* is a common habitant in freshwater bodies. *Legionella* already colonized in very low abundance the host before the symbiotic animals was collected for laboratory culturing. The aposymbiotic animals were later generated from these same animals using [3-(3,4-dichlorophenyl)-1,1-dimethylurea] (DCMU) treatment and were maintained as a separate culture for at least over 20 years (Habetha et al., 2003). On the aposymbiotic hosts, in absence of algae, *Legionella* bacterium became a dominant colonizer possessing invasive properties that also enables it to transiently colonize the symbiotic hosts through horizontal transmission.

Markedly, the long-term co-culturing study revealed that transfer of *Legionella spp. Hvir* from aposymbiotic to symbiotic polyps resulted in a decreased growth rate of symbiotic polyps and an increased algal release, indicating considerable fitness costs for symbiotic animals. Interestingly, over a longer period of time, the spread of algal symbionts among the co-cultured polyps and subsequent recolonization of aposymbiotic animals with *Chlorella* algae lead to a restoration of the microbiome to the native state of monocultivated symbiotic animals. Although the mechanism allowing horizontal transmission remains to be uncovered, it was intriguing to observe that *Legionella spp. Hvir* migrates actively toward the symbiotic hosts (Supplementary Figure 2). We speculate that this chemotactic movement of a *Legionella* species toward the symbiotic *H. viridissima* is caused by secretory products of the algae. Supporting this view, earlier study has demonstrated that many *Legionella* species possess chemotaxis genes and also show swarming behavior (Appelt and Heuner, 2017). We conclude that our observations suggest complex interactions between the members of the *H. viridissima* metaorganism and that the *Chlorella* photobiont is involved in controlling invading bacteria and stabilizing the resident microbiota.

With the available experimental evidences, it is difficult to provide any mechanistic explanation underlying the observed phenomenon, a major limitation being not possessing *Legionella spp. Hvir* in the culture. There might be several factors at play that affect the observed trend in microbial composition, including horizontal transmission, other bacterial symbionts, but we cannot rule out the involvement of the algal symbionts in *Legionella* mitigation. It is known for the renowned squid-vibrio symbiotic system that the *Vibrio* bacteria show chemotaxis in response to the host-derived chitin oligosaccharides (Mandel et al., 2012). Similar chemotactic behavior has been observed for several species of *Rhizobium* bacteria toward the plant roots (Aroney et al., 2021). Similarly, we hypothesize that algal metabolites in *Hydra* might be actively attracting the invasive species such as *Legionella* and altering the colonization niche to stop its further spread like “lure-and-kill” mechanism.

*Legionella* bacteria are generally perceived as pathogenic bacteria and are responsible for various human diseases, commonly an inhabitant of freshwater bodies. We have reported here an existence of a symbiotic strain of *Legionella* whose pathogenicity varies between the hosts. After colonizing the symbiotic animals, there is a transient pathogenic effect of bacterium on host fitness, but prolonged colonization reduces the bacterial virulence.

**FIGURE 4:**
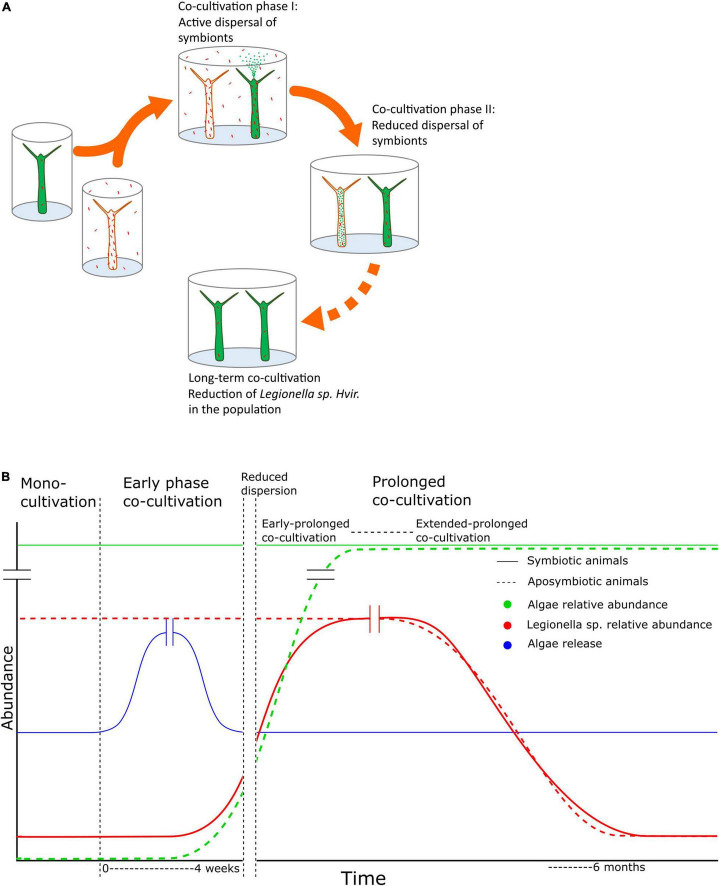
Summary figure. Summarized graphically in panels **(A,B)**, we represent the temporal dynamics of *Legionella spp. Hvir* in the population of aposymbiotic and symbiotic animals over a prolonged co-cultivation. There is a phase of active dispersal of symbionts during early co-cultivation followed by reduced dispersal. During this phase, the microbiome of both the strains changes over the course of time till the system reaches the phase of equilibrium in respect of alteration of microbiome composition and the entire population has a uniform microbial composition.

As shown in Figure 4, we present the dynamics of the bacterium in the population under the effect of horizontal transfer and the dominant symbiont, *Chlorella*. It is noteworthy that with the spread of algal symbionts in the population, the load of *Legionella spp. Hvir* decreases. Although the *Chlorella* photobiont and the bacterial microbiota colonize separate niches in the *H. viridissima* holobiont, the endosymbiotic algae can influence microenvironmental physicochemical parameters such as sugar, pH, and oxygen level and, thus, the properties of microbial habitat. Indeed, photosynthetic activity of *Chlorella* has been shown to involve transcriptional changes not only in the endoderm of *H. viridissima*, but also in the ectoderm that gives rise to the microbial habitat, the glycocalyx (Hamada et al., 2018). The fact that the spread of *Chlorella* symbiont correlates with *Legionella* mitigation in the community is consistent with an increased susceptibility in corals to white band disease post-bleaching event, resulting in the loss of immunity provided by the endosymbiotic algae (Muller et al., 2018). This transmission dynamics of symbionts would determine the outcome of the microbiome composition of different individuals in a population until it reaches the steady state. This modularity of the metaorganism, to alter the symbiont composition to adapt to the prevailing environment, makes the system resilient against adverse biotic or abiotic factors and improves the survival chances. This would also hold true for the natural conditions where the composition of a metacommunity is more complex. Collectively, these findings demonstrate the complexity of interactions in a tripartite symbiosis and the beneficial role of the *Chlorella* photobiont in maintaining a specific microbiome. This is of relevance because interhost dispersal of bacteria is not a rare phenomenon. Transmission of microbes from one individual to another one in a shared environment has been documented previously for aquatic systems, as exemplified in corals (Grupstra et al., 2021), zebrafish (Burns et al., 2017), microcosm (Shen et al., 2018), and zooplankton (Grossart et al., 2010). Moreover, co-housing of mice has a profound effect on their gut microbiome (Caruso et al., 2019; Robertson et al., 2019). It needs reductionistic model systems such as *Hydra* (Wittlieb et al., 2006; Augustin et al., 2012; Bosch, 2012; Murillo-Rincon et al., 2017; Hamada et al., 2018) for a functional understanding of the mechanisms involved.

## Materials and Methods

### Animal Husbandry

Experiments were carried out with the Australian *H. viridissima* strain A99, which was obtained from Dr. Richard Campbell, Irvine. In this study, the symbiotic hosts refer to the hosts with algal symbionts and the aposymbiotic hosts refer to the same animals without algal symbionts. However, both the lines of animals contain bacterial symbionts. Aposymbiotic polyps were obtained by photobleaching using 5 μM 3-(3,4-dichlorophenyl)-1,1-dimethylurea (DCMU) as described before (Habetha et al., 2003) and subsequently cultivated for more than 1 year prior to the start of the experiments. All the animals were cultivated at constant temperature (18°C), light conditions (12 h/12 h light/dark rhythm), and culture medium (0.28 mM CaCl_2_, 0.33 mM MgSO_4_, 0.5 mM NaHCO_3_, and 0.08 mM KCO_3_) according to the standard procedure (Lenhoff and Brown, 1970). For the co-cultivation experiments, symbiotic and aposymbiotic animals were co-cultivated and fed three times per week with freshly hatched *Artemia salina* for 4 weeks before DNA isolation. Before guide DNA (gDNA) extraction, polyps were starved for 72 h.

### Deoxyribonucleic Acid Extraction

For each biological replicate (*n* = 6), combined hydra and bacterial DNA were isolated from ten polyps. Animals were washed three times in sterile filtered (0.2 μm) culture medium and subsequently ruptured in buffer ATL (Qiagen, Germany) by vortexing. Algae were removed by centrifugation; suspensions were centrifuged three times at 350 *g* for 2 min. After each centrifugation step, supernatant was transferred to new tube. The gDNA was extracted from the third supernatant using the DNeasy Blood & Tissue Kit (Qiagen, Germany) with elution performed in 50 μl AE buffer. The extracted DNA was stored at −20°C until sequencing.

### 16S Ribosomal Ribonucleic Acid Gene Sequence Analysis

16S ribosomal RNA (rRNA) gene amplicon sequence analysis was conducted using the Quantitative Insights Into Microbial Ecology (QIIME) 1.8.0 package (Caporaso et al., 2010). Using the sequence FASTA file, a quality file, and a mapping file, which assigned the 10 nt barcodes to the corresponding sample as input, the sequences were analyzed using the following parameters: length between 300 and 400 bp, no ambiguous bases, and no mismatch to the primer sequence. Chimeric sequences were identified using ChimeraSlayer (Haas et al., 2011). We manually curated the chimeric sequences and as one of the criteria we considered those sequences that are present in at least two independent samples as false positive for being chimera. We considered it highly unlikely for an exact chimeric sequence to be present in two or more independent samples and, thus, decided to retain them. Sequences were rarified to the lowest number of reads in the dataset which for samples were just 2,300 reads. Such samples were accepted after manual curation through rarefaction plot and checking. Subsequently, sequences were grouped into operational taxonomic units (OTUs) at a ≥ 97% sequence identity threshold and mapped against SILVA_132 database (Quast et al., 2013).

### Migration Assay

As shown in Supplementary Figure 2A, 1 ml syringes were filled with 1 ml of sterile culture water. In the treatment group, 10 symbiotic polyps were placed in each syringe and the control syringes were filled with only water without polyps. These syringes were placed vertically in 1 L of water from the aposymbiotic animals (source pool). This source pool contained aposymbiotic animals for 1 week before the start of experiment and was removed during the experiment. The experiment was performed with six biological replicates, in six different setups, but the water for the source pool came from the same culture vessel to maintain equal bacterial load as the starter source. The syringes were incubated for 48 h in contact with the source pool water. At the end of incubation, the water in the syringes was carefully collected and used for DNA extraction and quantification.

### *Legionella spp. Hvir* Quantification Using Real-Time PCR

*Legionella*-specific primers were designed using the V1–V2 region sequences obtained from 16S rDNA sequencing: forward 5′-CTCTCAGACCAGCTACCGAT-3′ and reverse 5′-TACTAGATGGGTGGCGAGTG-3′. They were checked by aligning against the Ribosomal Database Project (RDP) database using the RDP probe (Cole et al., 2014) and against the 16S rRNA library of *H. viridissima* to avoid any non-specific amplification. For the migration assay, 1 ml of water was collected from the syringe or the source pool (Supplementary Figure 2A) and centrifuged at 20,000 rcf for an hour and a half. A total of 950 μl of the supernatant was removed away to avoid any disturbance in the pellet. DNA was isolated from the remaining 50 μl water + pellet using the DNeasy Blood & Tissue Kit (Qiagen, Germany) with elution performed in 25 μl AE buffer. Real-time PCR was performed on 1 μl of the template DNA using the GoTaq qPCR Master Mix (Promega, Madison, WI, United States) and ABI Prism 7300 (Applied Biosystems, Foster City, CA, United States). The qPCR was performed in duplicate with six biological replicates each.

### Fractionation of Water From Aposymbiotic Animals

The aposymbiotic animals were incubated in sterile S-medium for 4 days without feeding. After 4 days, this medium was used to prepare three different fractions containing the bacteria/viruses/metabolites. The medium was centrifuged at 20,000 *g* for 30 min to collect bacteria in the pellet. The pellet was resuspended in a smaller volume of sterile S-medium. The supernatant was filtered through a sterile 0.2 u filter to remove bacterial particles. Half of the supernatant was used to collect viral particles and other half for metabolite fraction. One half of the supernatant was filtered through 0.02 u filter to remove viral particles and obtain metabolite fraction. Polyethylene glycol (PEG) was added to rest of the supernatant suspension to achieve the final concentration of 5% to precipitate viral particles. The mixture was centrifuged at 20,000 *g* for 75 min. The supernatant was carefully discarded and the pellet was resuspended in sterile S-medium. The animals were incubated in each of the water fraction for 48 h before DNA isolation for 16S rRNA sequencing. All the samples were treated using the same water source and collected simultaneously to avoid any additional variation and contamination.

### Algal Release Quantification

For early co-cultivation, monocultivated symbiotic polyps were co-cultivated with monocultivated aposymbiotic polyps for 48 h prior to start of experiment. For prolonged co-cultivation, the animals were co-cultivated for 1 month. At 0 h before the start of experiment, four symbiotic polyps of monocultivated animals or four polyps each of symbiotic and aposymbiotic animals, each of early/prolonged co-cultivation, were incubated in 1 ml of sterile culture water (Supplementary Figure 5). As an additional animal-density control, 8 polyps of monocultivated symbiotic animals were used (Supplementary Figure 5) to match the co-cultivated animals. After 24 h, 900 μl of water was carefully collected and centrifuged at 14,000 rpm for 5 min to pellet the algae. A total of 850 μl of the supernatant was carefully removed away and the pellet was resuspended in the remaining 50 μl of water. It was immediately subjected to counting using flow cytometry on the BD FACSCalibur with CellQuestPro v5.2 (*Becton–Dickinson*) with a blue laser at 488 nm using forward scatter and FL3 filter (>670 nm). The data were further analyzed with FCSalyzer 0.9.13-alpha^1^ and total number of algal cells expelled per polyp per ml of water was calculated.

## Data Availability Statement

The datasets presented in this study can be found in online repositories. The names of the repository/repositories and accession number(s) can be found below: Sequence Read Archive (SRA), accession no: PRJNA810523.

## Author Contributions

JB, KS, SF, TL, and TB planned the experiments. JB, KS, and TL executed the experiments. PR provided cell counting facility and logistics. JB analyzed the data. JB, SF, and TB wrote, reviewed, and edited the manuscript. All authors contributed to the article and approved the submitted version.

## Conflict of Interest

The authors declare that the research was conducted in the absence of any commercial or financial relationships that could be construed as a potential conflict of interest.

## Publisher’s Note

All claims expressed in this article are solely those of the authors and do not necessarily represent those of their affiliated organizations, or those of the publisher, the editors and the reviewers. Any product that may be evaluated in this article, or claim that may be made by its manufacturer, is not guaranteed or endorsed by the publisher.

## Footnotes

1
https://sourceforge.net/projects/fcsalyzer/
