# Supplementary material for: Symbiotic Algae of Hydra viridissima Play a Key Role in Maintaining Homeostatic Bacterial Colonization
Source: Front Microbiol. 2022 Jun 6;13:869666. doi: 10.3389/fmicb.2022.869666 (PMC9207534; doi:10.3389/fmicb.2022.869666)

Supplementary table 1:

The table comprises of the relative abundance of *Legionella sp. Hvir* in all the samples of symbiotic and aposymbiotic animals under various cultivation conditions and treatments. The table also contains the respective treatment or experimental condition.

Supplementary table 2:

The table contains the results of Adonis test comparing the symbiotic and aposymbiotic animals under mono- and co- cultivation using Bray-Curtis dissimilarity matrix as input.

Supplementary table 3:

The table contains the results of Adonis test comparing the symbiotic animals treated with various fractions of water from the culture of aposymbiotic animals using Bray-Curtis dissimilarity matrix as input.

Supplementary table 4:

The table contains the results of Adonis test comparing the symbiotic and aposymbiotic animals under mono- or co-cultivation and transfer of symbiotic animals from co- to mono-cultivation, using Weighted-Unifrac distance matrix as input.

Supplementary figure 1:

Absolute quantification of *Legionella sp. Hvir* in aposymbiotic and symbiotic animals under mono-cultivation. (Statistical test: Student's t-test, n=4, ** p-value<0.01)

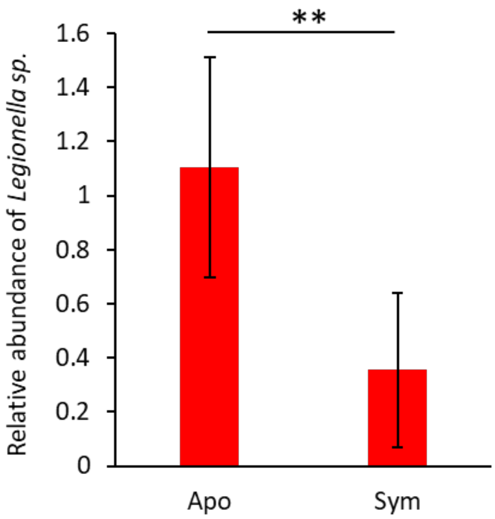

Supplementary figure 2:

A Experimental setup for the migration assay (see materials and methods). The treatment syringe held symbiotic polyps and the control syringe was without polyps. The source pool is the water from aposymbiotic animals. **B** qPCR with *Legionella sp. Hvir* specific primers showed an increased migration of *Legionella* in the treatment syringe as compared to the control (n=6, t-test, *p<0.05, ***p<0.001).

A **B**

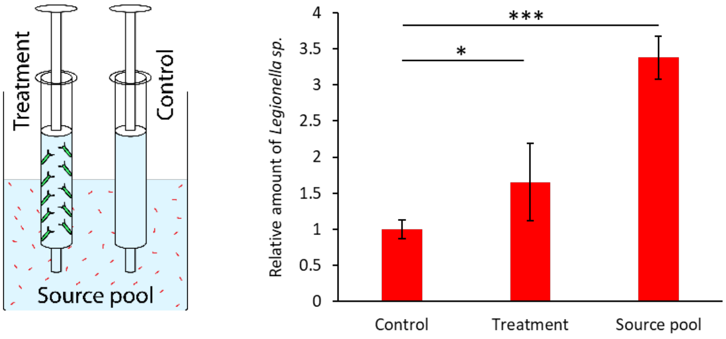

Supplementary figure 3:

Relative abundance of *Legionella sp. Hvir* (normalized to total bacteria) in the symbiotic animals in response to the bacterial/viral/metabolite fraction of the culture water from the aposymbiotic animals. The Y axis represent the relative abundance and the X axis represent the fraction received by the symbiotic animals from the culture water of aposymbiotic animals, compared with the mono-cultivated symbiotic animals (Sym_mono). The red bars indicate the relative abundance in each biological replicate and the blue bar indicates the average relative abundance.

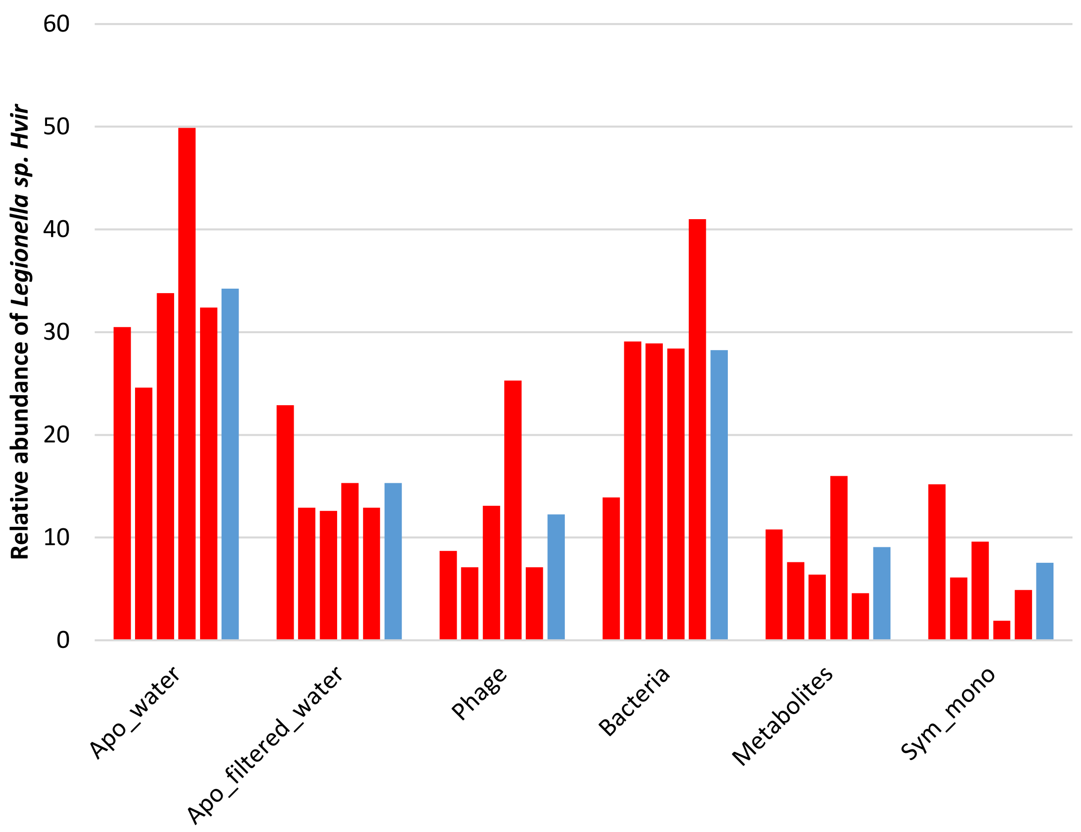

Supplementary figure 4:

The co-cultivated aposymbiotic animals showed a reduced growth rate that can be accounted by establishment of new symbiosis with the expelled algae from the symbiotic host.

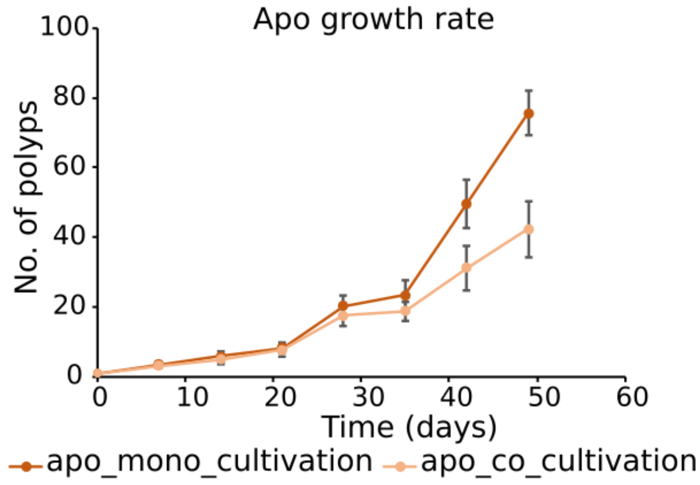

Supplementary figure 5:

Experimental setup for algal expulsion measurement. Each tube contained 1 ml of culture water. Each tube contained 4 polyps (x4), 8 polyps (x8) or 8 polyps (4 each of symbiotic and aposymbiotic polyps from early/prolonged co-cultivation) 900 ml of water was collected and subjected to centrifugation at 14,000 rpm for 5 min followed by removal of 850 µl of water. The pellet was resuspended in remaining 50 µl of water and used for counting the total number of algal cells.

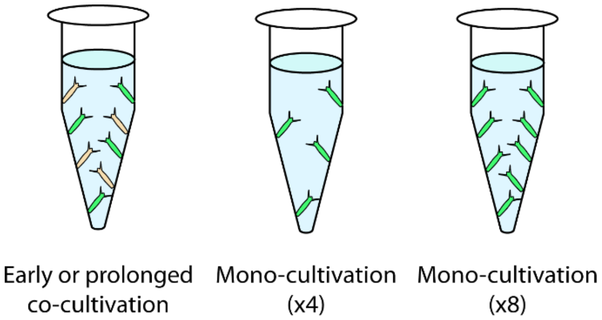

Supplement: Supplementary file 5 [file Data_Sheet_1.PDF]
